# The Tangential Extraperitoneal Retrorenal Approach in Kidney Transplant Biopsy: An Observational Study to Assess Complication and Adequacy Rates

**DOI:** 10.3389/ti.2021.10068

**Published:** 2022-01-13

**Authors:** Markus Pirklbauer, Martin Berger, Miro D. Boban, Martin Tiefenthaler

**Affiliations:** Department of Internal Medicine IV – Nephrology and Hypertension, Medical University Innsbruck, Innsbruck, Austria

**Keywords:** percutaneous kidney transplant biopsy, tangential extraperitoneal retrorenal approach, ultrasound-guided biopsy, complication rate, adequacy rate

## Abstract

**Introduction:** Ultrasound-guided percutaneous kidney allograft biopsy is the gold-standard for pathology work-up. Recent studies postulate better safety and efficacy for tangential approaches, however, there is no recommendation regarding biopsy needle path. In this context, we previously described the unified tangential extraperitoneal retrorenal (TER) approach for standard allograft biopsy.

**Methods:** A single-center retrospective observational study evaluated safety and efficacy of the TER biopsy approach among 250 patients that underwent 330 ultrasound-guided kidney transplant biopsies between January 2011 and May 2020.

**Results:** The overall major complication rate was 0.56% per biopsy attempt (1.21% per biopsy) including blood transfusion, arterial embolization and bladder catheterization for gross hematuria in 0.28, 0.14 and 0.14% of biopsy attempts, respectively (0.61, 0.30 and 0.30% of biopsies, respectively). Minor complications included subcapsular and/or perinephric hematoma, superficial bleeding, arteriovenous fistula and gross hematuria in 12.6, 3.0, 2.5 and 1.4% of biopsy attempts, respectively (27.0, 6.4, 5.5 and 3.0% of biopsies, respectively). Sample adequacy rate was 86.7%, ranging from 82.2 to 94.1% if one or ≥two cores were analyzed, respectively. Residents and consultants yielded similar complication and adequacy rates.

**Conclusion:** According to current literature, ultrasound-guided TER kidney transplant biopsy is a safe and efficient approach eligible for nephrology training.

## Graphical Abstract

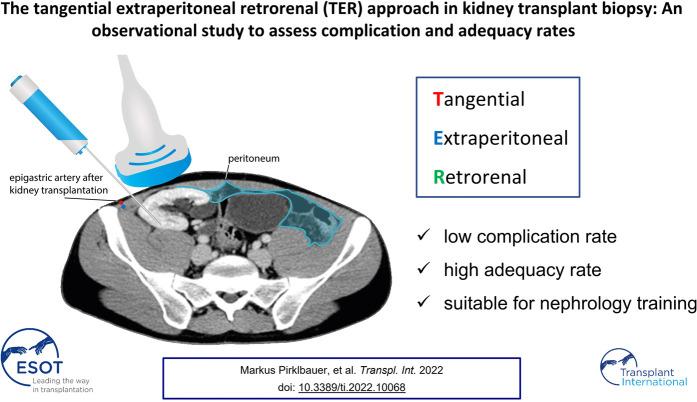

## Introduction

Ultrasound-guided percutaneous renal transplant biopsy is the gold-standard procedure for allograft pathology work-up. Recent studies, including previous research at our institution, postulate better safety and efficacy of tangential compared to radial approaches [1–3], however, there is no general consensus regarding biopsy needle path for this standard technique. A tangential biopsy allows to direct the needle tip away from the renal hilum, the ureter, and large vessels of the anastomosis region, thereby sparing these anatomical structures from potential injury. In this regard, we recently developed the so called tangential, extraperitoneal, retrorenal (TER) approach for standard allograft biopsy, that penetrates the allograft parallel to the renal capsule (tangential component, T), keeps safe distance to the peritoneal fold (extraperitoneal component, E), and targets the posterior side of the allograft (retrorenal component, R) in a lateral-to-medial approach. A pilot study among 104 patients already demonstrated excellent safety and efficacy of the TER approach in 127 kidney transplant biopsies [1]. In our present study we verify these results in a larger patient cohort by demonstrating excellent complication and adequacy rates among 250 patients undergoing 330 kidney transplant biopsies utilizing a conventional (96.1%) or modified (3.9%) TER approach. Furthermore, this is the first study to 1) assess both major and minor complications based on a standardized post-procedural ultrasound follow-up as well as to 2) confirm the eligibility of TER kidney transplant biopsy for nephrology training.

## Patients and Methods

A single-center retrospective observational study was conducted at our Department to assess safety and efficacy of TER kidney transplant biopsy. Between January 2011 and May 2020, 250 patients underwent at least one kidney transplant biopsy at our institution and were included in the present study. The TER approach is the standard technique for kidney transplant biopsy at our institution and was performed in 317/330 allograft biopsies (96.1%). A modified TER approach, which featured only two of the three components of the conventional TER approach (tangential, extraperitoneal, retrorenal), had to be conducted in 13/330 biopsies (3.9%) due to anatomical causes, e.g., dislocated inferior epigastric artery, orthotopic allograft transplantation or preexisting hematoma. 104 of 250 study patients were already included in our previous pilot study [1]. 6/330 allograft biopsies (1.8%) were protocol biopsies, the remaining 324 biopsies were based on indication. Patient data were available from the institutions’ computerized clinical documentation systems.

### Biopsy Protocol

Kidney transplant biopsy was exclusively performed in an inpatient setting where patients are admitted to hospital on the day of biopsy and discharged on the following day. Anti-platelet/anti-coagulant medication was halted from 7–14 days prior to 7–14 days after biopsy depending on the type of drug. Patients at high risk of thromboembolism were administered enoxaparin-sodium during that period; however, enoxaparin-sodium was administered no later than 24 h before biopsy. Blood pressure and heart rate were monitored peri-interventionally. Anti-hypertensive medication (e.g., nitroglycerine and/or urapidil and/or dihydralazine) was administered if blood pressure peri-interventionally exceeded 160/90 mmHg. Lorazepam sedation was available for episodes of anxiety and/or agitation; however, patients did not receive general anesthesia. Following biopsy, patients had to remain in a supine position and use an abdominal belt to minimize the risk of hematoma. Monitoring ended 5 h after biopsy if a post-biopsy urine void and no signs of gross hematuria, flank pain or other symptoms indicating a complication were reported. A blood count as well as color-duplex ultrasound examination was performed on the next day to detect bleeding complications or arterio-venous fistulas (AVF). If both examinations yielded normal findings, patients were discharged from hospital and instructed to avoid weight-lifting >5 kg and contact sports for 14 days. Normal physical activity including running and cycling was encouraged to prevent thromboembolism. Patients were asked to immediately return to the hospital in case of discomfort after discharge.

### Biopsy Technique

**FIGURE 1:**
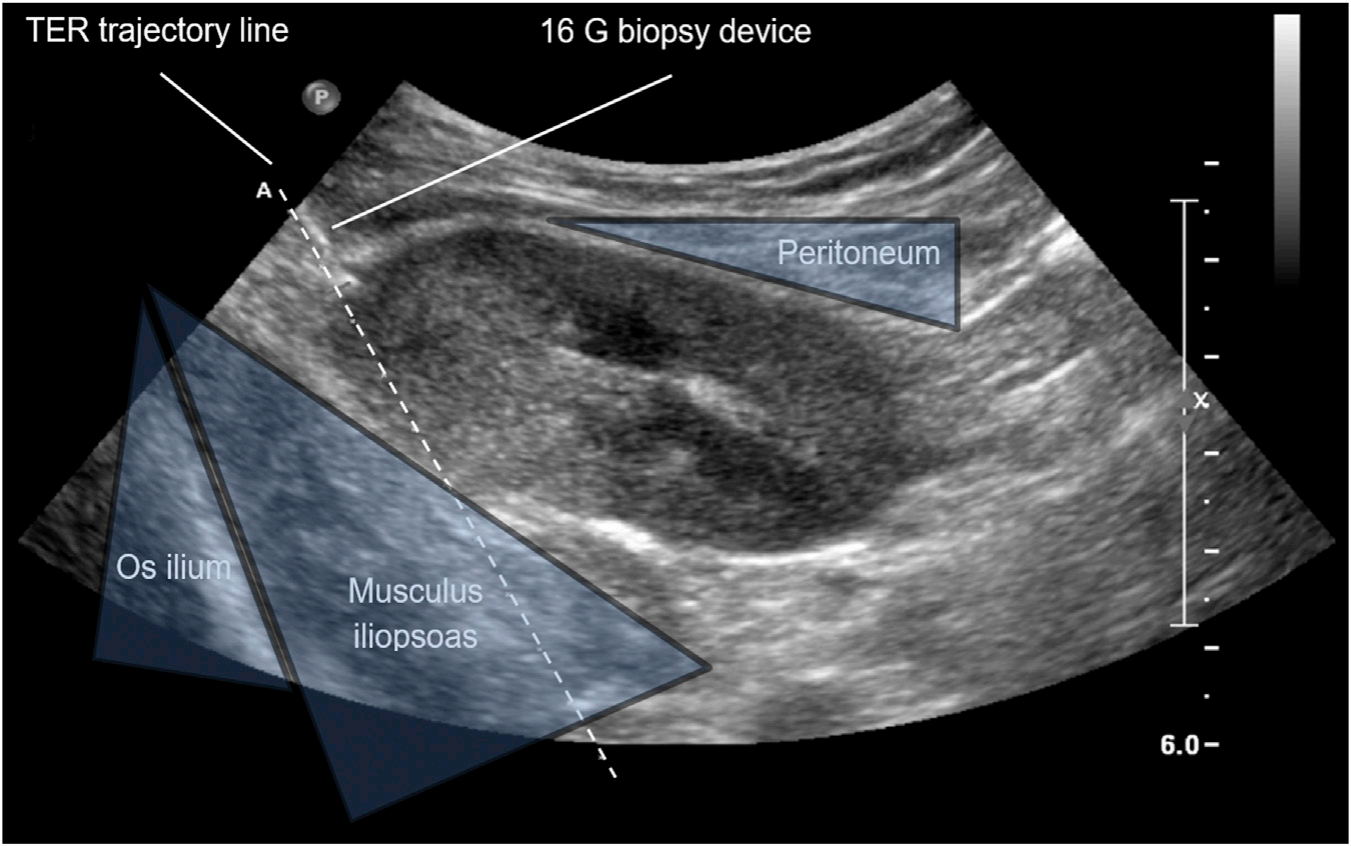
Anatomic landmarks of real-time ultrasound-guided kidney allograft biopsy. Ultrasound image of the right iliac kidney allograft; TER, tangential, extraperitoneal, retrorenal; G, gauge.

**FIGURE 2:**
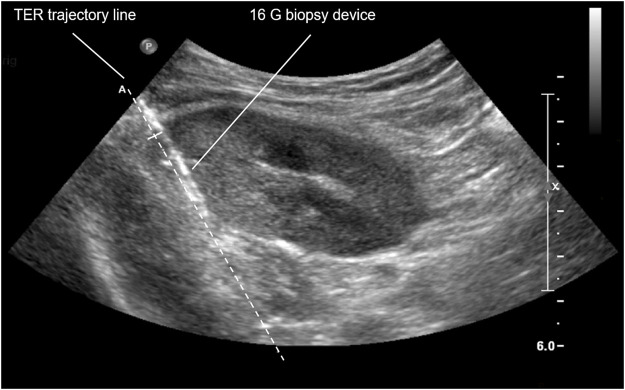
TER biopsy of right iliac kidney allograft. TER, tangential, extraperitoneal, retrorenal; G, gauge.

Real-time ultrasound-guided TER kidney transplant biopsy is performed in supine or—in case of obesity or pendulous abdomen—lateral decubitus position. A needle guidance system mounted to the ultrasound transducer optimizes needle handling and helps to visualize needle path. The ultrasound transducer is placed approximately 2 cm medial to the anterior superior iliac spine to determine optimal biopsy area. The latter allows to 1) penetrate the allograft parallel to the renal capsule (i.e., tangential component), 2) keep safe distance to the peritoneal fold (extraperitoneal component), and 3) target the posterior side of the upper pole or the most dorsal part of the lateral portion of the allograft (retrorenal component) (Figure 1). Local anesthesia with xylocaine 2% is administered as subcutaneous depot prior to skin incision as well as ultrasound-guided deep depot along the needle path up to the renal capsule. *Via* a small skin incision the biopsy device is then advanced towards the allograft from lateral to medial in a transverse plane using real-time ultrasound guidance. Once the renal capsule is reached, the biopsy needle is fired tangentially into the outer third of the renal cortex (Figure 2). Bedside analysis of biopsy cores for adequacy was routinely done by using a magnifying glass for the crude assessment of glomerular number. Whenever feasible, at least two core samples measuring 1.3 mm in diameter and 22 mm in length are obtained using a 16 cm long, 16 Gauge (G) spring-loaded biopsy device (Bard^®^ Monopty^®^ Disposable Core Biopsy Instrument).

### Definition of Complications

Major complications were defined as biopsy-related complications requiring invasive therapy and included bladder catheterization for gross hematuria, blood transfusion (following either a biopsy-related drop of hemoglobin or image confirmation of biopsy-related bleeding), interventional radiology procedure with or without arterial embolization, surgery, graft loss, or death. Minor complications were defined as any biopsy-related relevant medical condition not requiring invasive therapy. Complication rates were calculated per biopsy attempt and biopsy event.

### Definition of Adequacy

According to the criteria of the Banff 97 working classification of renal allograft pathology [4], a biopsy core sample was considered 1) adequate if it contained at least 10 glomeruli and two arteries or 2) minimal if it contained a minimum of seven glomeruli and one artery in the pathologist`s assessment. Adequacy rates given in the present study represent the sum of samples deemed either minimal or adequate. Adequacy was calculated per biopsy as glomerular and arterial yield were reported per biopsy only.

### Statistical Analysis

Descriptive statistics was performed using Microsoft Excel (Microsoft Corporation, Redmond, Washington, United States). Results and baseline characteristics are presented as absolute frequencies or mean values ± standard deviation (range). Chi^2^ statistics was performed with SPSS version 24.0 to assess potential associations between nominal parameters (i.e., training status, occurrence of complications and sample adequacy). The level of significance (*p* value) was set to 0.05.

### Statement of Ethics

The study was conducted in accordance with the World Medical Association Declaration of Helsinki. The study protocol was reviewed and approved by the Innsbruck Medical University ethics committee prior to study initiation (approval number ECS 1106/2020). Patient information was managed entirely coded. All patient associated data are subject to privacy protection according to the current European General Data Protection Regulation. Based on the retrospective study design the Innsbruck Medical University ethics committee granted an exemption from requiring written informed consent.

## Results

### Patient and Biopsy Characteristics

330 ultrasound-guided kidney transplant biopsies were performed among 250 patients between January 2011 and May 2020. 203 (61.5%) and 127 (38.5%) biopsies were performed on male and female patients, respectively. 194 (77.6%) patients underwent one biopsy, however, patients were subjected to kidney transplant biopsy up to five times. 2, 3, 4 and 5 biopsies were performed in 39 (15.6%), 11 (4.4%), 5 (2.0%) and 1 (0.4%) patient, respectively. Median age and body mass index at the time of biopsy was 50 years (range 18–78) and 24.3 (range 15.6–42.0), respectively. The mean number of kidney transplants per patient was 1.41 ± 0.8 (range 1–7). The total number of biopsy attempts was 709, yielding 637 core samples. Mean number of biopsy attempts per biopsy and recovered core samples was 2.2 ± 0.6 (range 1–5) and 1.9 ± 0.5 (range 1–5), respectively. TER biopsy was performed in 317/330 biopsies (96.1%) and 683/709 biopsy attempts (96.3%). In 13 biopsies (3.9%) and 26 biopsy attempts (3.7%), a modified TER approach had to be applied due to anatomical causes, e.g., dislocated inferior epigastric artery, orthotopic allograft transplantation or preexisting hematoma. Tangential, extraperitoneal or retrorenal biopsy could not be performed in four, two and seven biopsies, respectively. Though, at least two components of the conventional TER approach were performed in these 13 cases (Tables 1, 2).

**TABLE 1:** Patient characteristics.

Patients	250
Male	155 (62.0)
Female	95 (38.0)
No. of performed biopsies	330
On male patients	203 (61.5)
On female patients	127 (38.5)
Per patient	
1	194 (77.6)
2	39 (15.6)
3	11 (4.4)
4	5 (2.0)
5	1 (0.4)
No. of kidney transplant	1.41 ± 0.8 (1–7)
Age (years)	50 (18–78)
Body Mass Index (kg/m^2^)	24.3 (15.6–42.0)
Arterial hypertension (≥140/90 mmHg)	291 (88.2)
Diabetes mellitus	76 (23.0)
Arterial hypertension and diabetes mellitus	69 (20.9)

Data are presented as numbers (percent), mean ± standard deviation (range), or median (range) for age and body mass index.

No., number.

**TABLE 2:** Biopsy characteristics.

No. of performed biopsies	330
No. of biopsy attempts per biopsy	
1	28 (8.5)
2	237 (71.8)
3	57 (17.3)
4	4 (1.2)
5	4 (1.2)
No. of biopsy attempts	2.2 ± 0.6 (1–5)
Total	709
No. of samples recovered	1.9 ± 0.5 (1–5)
Total	637 (89.9)
No. of biopsy attempts without recovery of sample	72 (10.2)
Biopsy technique	
No. of performed biopsies	330
Using TER approach	317 (96.1)
Using modified TER approach	13 (3.9)
No. of biopsy attempts	709
Using TER approach	683 (96.3)
Using modified TER approach	26 (3.7)

Data are displayed as number (percent) or mean ± standard deviation (range).

ND, no data; No., number.

### Major Complications

Among 709 biopsy attempts (330 biopsies), four major complications (0.6% of biopsy attempts and 1.2% of biopsies) were documented among 3 patients throughout the study period. Considering conventional TER approach only with 683 biopsy attempts (317 biopsies), 2 complications (0.3% of biopsy attempts and 0.6% of biopsies) were classified as major complications. One patient was subject to rinsing catheterization of the bladder (0.1% of biopsy attempts and 0.3% of biopsies) due to gross hematuria following biopsy. Transfusion of blood products (0.3% of biopsy attempts and 0.6% of biopsies) was required in two patients. One of these patients underwent conventional TER kidney transplant biopsy and received two units of packed red blood cells on the day after biopsy on account of suspected bleeding in abdominal ultrasound examination and CT scan. The other patient underwent a modified TER approach (radial biopsy) and experienced aggravated pain immediately after biopsy. Instant ultrasound examination revealed arterial bleeding involving the upper pole renal artery. Emergency coiling (0.1% of biopsy attempts and 0.3% of biopsies) was conducted and four units of packed red blood cells and platelet concentrates were administered for low hemoglobin and platelet count. No patient required surgical treatment. No graft losses or deaths occurred (Table 3).

**TABLE 3:** Biopsy complications.

	TER + modified TER		TER only	
	709 biopsy attempts	330 biopsies	683 biopsy attempts	317 biopsies
Overall				
Minor	149 (21.0)	149 (45.2)	141 (20.6)	141 (44.5)
Major	4 (0.6)	4 (1.2)	2 (0.3)	2 (0.6)
Total	153 (21.6)	153 (46.4)	143 (20.9)	143 (45.1)
Periprocedural minor complications				
Drainage of serous fluid	2 (0.3)	2 (0.6)	2 (0.3)	2 (0.6)
Vasovagal reaction	5 (0.7)	5 (1.5)	5 (0.7)	5 (1.6)
Hypertensive urgency	1 (0.1)	1 (0.3)	1 (0.2)	1 (0.3)
Superficial bleeding	21 (3.0)	21 (6.4)	19 (2.8)	19 (6.0)
Postprocedural minor complications				
Gross hematuria	10 (1.4)	10 (3.0)	10 (1.5)	10 (3.2)
Arteriovenous fistula	18 (2.5)	18 (5.5)	18 (2.6)	18 (5.7)
Subcapsular hematoma	7 (1.0)	7 (2.1)	6 (0.9)	6 (1.9)
Perinephric hematoma	82 (11.6)	82 (24.9)	78 (11.4)	78 (24.6)
<3 × 1 cm	69 (9.7)	69 (20.9)	66 (9.7)	66 (20.8)
>3 × 1 cm	8 (1.1)	8 (2.4)	7 (1.0)	7 (2.2)
ND	5 (0.7)	5 (1.5)	5 (0.7)	5 (1.6)
Pain^a^	2 (0.3)	2 (0.6)	1 (0.2)	1 (0.3)
Deep vein thrombosis	1 (0.1)	1 (0.3)	1 (0.2)	1 (0.3)
Major complications				
Rinsing catheter for gross hematuria	1 (0.1)	1 (0.3)	1 (0.2)	1 (0.3)
Transfusion	2 (0.3)	2 (0.6)	1 (0.2)	1 (0.3)
Coiling/arterial embolization	1 (0.1)	1 (0.3)	0 (0.0)	0 (0.0)

a No ultrasound correlate.

Data are displayed as number (percent).

ND, no data; No., number.

### Minor Complications

Overall, 149 events were classified as minor complications (21.0% of biopsy attempts and 45.2% of biopsies). Considering TER approach only, 141 minor complications were documented (20.6% of biopsy attempts and 44.5% of biopsies). Routine ultrasound examination on day 1 after kidney transplant biopsy identified 82 perinephric hematomas, eighteen AVF, and seven subcapsular hematomas (i.e., 11.6, 2.5 and 1.0% of biopsy attempts, respectively and 24.9, 5.5 and 2.1% of biopsies, respectively). Of the perinephric hematomas, 69 were smaller than 3 × 1 cm, eight were bigger than 3 × 1 cm and five could not be categorized because of missing data (i.e., 9.7, 1.1 and 0.7% of biopsy attempts, respectively and 20.9, 2.4 and 1.5% of biopsies, respectively). All AVF had resolved spontaneously at follow-up ultrasound examination. 21 superficial bleedings, 10 episodes of gross hematuria, 5 vasovagal reactions requiring atropine administration, and one hypertensive urgency requiring administration of urapidil, dihydralazine and amlodipine, were detected after kidney allograft biopsy (i.e., 3.0, 1.4, 0.7, and 0.1% of biopsy attempts, respectively and 6.4, 3.0, 1.5 and 0.3% of biopsies, respectively). Abdominal pain (0.3% of biopsy attempts and 0.6% of biopsies) was reported in two patients. In both cases, no ultrasound correlate was detected and both patients were administered analgesic medication. One case of deep vein thrombosis (0.1% of biopsy attempts and 0.3% of biopsies) of the ipsilateral popliteal vein was documented (Table 3).

### Sample Adequacy

Cores samples were evaluated according to the Banff 97 working classification of renal allograft pathology [4]. 192 (58.2%) and 94 (28.5%) biopsies were considered adequate and minimal, respectively. Thus, a total of 286 biopsies (86.7%) met the criteria for sample adequacy. 42 biopsies (12.7%) were considered inadequate and data from two biopsies (0.6%) were lacking. Adequacy rate increased to 94.1%, if two or more core samples were analyzed. Mean number of glomeruli and arteries was 15.7 ± 9.3 glomeruli (range 0–69) and 2.5 ± 1.6 arteries (range 0–10), respectively (Table 4).

**TABLE 4:** Sample adequacy.

No. of performed biopsies	330
No. of analyzed samples	
0	2 (0.6)
1	174 (52.7)
2	150 (45.5)
3	2 (0.6)
ND	2 (0.6)
No. of biopsies considered	
Adequate	192 (58.2)
Minimal	94 (28.5)
Inadequate	42 (12.7)
ND	2 (0.6)
Adequate and minimal	286 (86.7)
If 1 sample analyzed	143 (82.2)
If 2 samples analyzed	141 (94.0)
If 3 samples analyzed	2 (100.0)
If 2 or 3 samples analyzed	143 (94.1)
No. of glomeruli	15.7 ± 9.3 (0–69)
No. of arteries	2.5 ± 1.6 (0–10)

Data are displayed as number (percent) or mean ± standard deviation (range).

ND, no data; No., number.

### Complications and Sample Adequacy of Training Biopsies

116 (35.2%) and 214 (64.9%) biopsies were performed by nephrology residents and consultants, respectively. Major and minor complications occurred in 1.2 and 20.5% of resident biopsy attempts, respectively (2.6 and 44.0% of resident biopsies, respectively) and 0.2 and 21.3% of consultant biopsy attempts, respectively (0.5 and 45.8% of consultant biopsies, respectively). *p* = 0.094 and *p* = 0.798 for association of resident status with major and minor complications, respectively. Considering TER approach only, major complication rate per biopsy attempt was 0.4 and 0.2% among 249 (35.1%) resident and 460 (64.9%) consultant biopsy attempts, respectively (i.e., 0.9 and 0.5% among 111 resident and 206 consultant biopsies, respectively). Overall adequacy rate was 87.9 and 86% for biopsies performed by residents and consultants, respectively (*p* = 0.619 for association of resident status with sample adequacy) (Supplementary Tables S1, S2)*.*

## Discussion

The present study reinforces the results of a recent pilot study [1] demonstrating excellent safety and efficacy of TER kidney transplant biopsy and corroborates previous findings showing low major complication and high adequacy rates with the use of tangential kidney allograft biopsy [2, 3, 5]. With a major complication rate of 0.3% per biopsy attempt (0.6% per biopsy) the TER approach is among the safest allograft biopsy approaches according to current literature (Supplementary Table S3). Major complication rates have been previously demonstrated to be up to 5.6% [6]; however the latter study did not report a specific biopsy region or needle path. Comparable studies utilizing a tangential biopsy technique reported major complication rates ranging between 0.0% [5] and 3.6% [7]. While the former study first described the so called “cortex-only” view among 188 biopsies, the latter study used a computer tomography (CT)-guided approach among 28 biopsies. While small patient number is a substantial limitation of both studies, CT-guided approaches implicate additional risk from radiation exposure. The most comprehensive studies assessing ultrasound-guided tangential allograft biopsy yielded major complication rates of 0.7 [2], 0.3 [3] and 1.9% [8]. Minor complications, such as AVF and hematomas, are best detected through standardized post-procedural ultrasound examination and/or blood count; however, most of the available studies, including comparable studies with tangential biopsy techniques [2, 3, 5, 8], did not routinely perform post-procedural ultrasound and/or blood count. Thus, it is likely to speculate that these studies might not reflect the true incidence of minor complications. By performing routine ultrasound and blood count on the day after biopsy, our study is the first to comprehensively assess both symptomatic and asymptomatic complications. Based on these substantial differences in post-procedural management, however, the minor complication rates found in the present study are not comparable to previous studies in the field. AVF are usually asymptomatic and rarely require specific therapy; however, centers performing ultrasound-based screening report AVF rates of up to 10.7% [9]. Generally, AVF rate seems to correlate with both the extent and timing of post-procedural ultrasound examination. Consequently, AVF rates are usually reported to be low in studies that do not routinely perform post-procedural imaging [7, 10–13] and tend to be higher in studies that perform ultrasound examination within hours [9, 14, 15] as compared to those performing immediate post-procedural ultrasound [16–18].

The low AVF rate (2.5% per biopsy attempt and 5.5% per biopsy) found with the TER biopsy approach is likely to result from targeting the outer third of cortical renal parenchyma and thus, from sparing larger vessels in the medullary region. All AVF spontaneously resolved at a 2 weeks follow-up examination. Nevertheless, screening might be beneficial for individual patients as AVF-associated severe complications, such as arterial embolization and nephrectomy have been described in the literature [10, 15]. A recent retrospective Japanese study described an AVF rate of 2.6% after kidney allograft biopsy and proposed that embolization is a safe treatment for these AVF. However, the authors state that the study was likely to underestimate AVF rate as post-procedural management was not consistent among the study population [19].

As for AVF, hematoma detection rate correlates with the availability of post-procedural ultrasound examination and ranges from 0.0% in studies that did not perform ultrasound examination [20] and 13.4% [21] in studies that performed immediate postprocedural ultrasound. With a consequent post-procedural ultrasound examination, the present study reports an overall hematoma rate of 12.6% per biopsy attempt (27.0% per biopsy). A previous study performing comparable post-procedural management reported a similar hematoma rate of 11.1% [22]. While none of the reported perinephric (11.6% of biopsy attempt and 24.9% of biopsies) and subcapsular (1.0% of biopsy attempts and 2.1% of biopsies) hematomas required specific therapy in our study, individual patients might benefit from hematoma screening as large hematomas may profit from extended period of rest or—if applicable—extended period of anti-platelet/anticoagulant withdrawal post biopsy. Additionally, post-procedural screening might help to timely identify large hematomas that will require surgical evacuation in order to preserve kidney allograft function [6, 8, 13]. Nevertheless, substantial heterogeneity exists in post-procedural management of kidney transplant biopsy between facilities, partly due to reimbursement issues. While overnight in-hospital observation is clinical routine in many European and Japanese centers, others, including most U.S. facilities, perform shorter observation periods. In this regard, a recent study by Patel et al. both corroborated the low rate of major bleeding complications with ultrasound-guided renal transplant biopsy (0.2%) and presented evidence that a standardized 1-hour postprocedure observation protocol can be safely used. However, the authors state that more than half of these complications were not clinically apparent within 4 h of biopsy [23]. Overnight in-hospital observation is part of the routine post-procedural management at our facility, however, the present study does not advocate any specific post-procedural management strategy at this time.

The occurrence of gross hematuria following kidney allograft biopsy ranges from 0.0% [24] and 9.0% [13] in the literature. Our finding of a rather low gross hematuria rate (1.4% per biopsy attempt and 3.0% per biopsy) is consistent with the low rate (0.7%) found in another tangential allograft biopsy study [2]. Other minor complications, such as superficial bleedings, vasovagal reactions, hypertensive urgency, seroma drainage, and aggravated pain, are rarely reported in the literature, and thus, occurrence rates are difficult to compare. Deep vein thrombosis that is normally associated with immobilization might not be considered as direct biopsy complication. Previous studies reported that renal allograft biopsy within 30 days after transplantation, deep puncture (i.e., high percentage of medulla) and the number of biopsy attemps per biopsy increase the risk of AVF [25, 26]. In our study, 47/330 biopsies (14%) were performed within 30 days after kidney transplantation, however, we did not find a significant association with major and/or minor complications. Interestingly, the latter did also not significantly correlate with the number of biopsy attempts per biopsy. While major complications exclusively occurred among patients that were subject to 2 biopsy attempts, AVF rate did not increase with the number of biopsy attempts per biopsy (up to 5). Albeit not statistically significant, hematoma rate nominally increased from 25% (with up to 4 biopsy attempts) to 50% (with 5 biopsy attempts) (*p* = 0.24). Data regarding the percentage of medulla in biopsy specimen is not available for the present study, however, deep puncture would be a rare finding with adequate TER biopsy as parallel orientation of the biopsy needle to the renal capsule should avoid any deep puncture.

**FIGURE 3:**
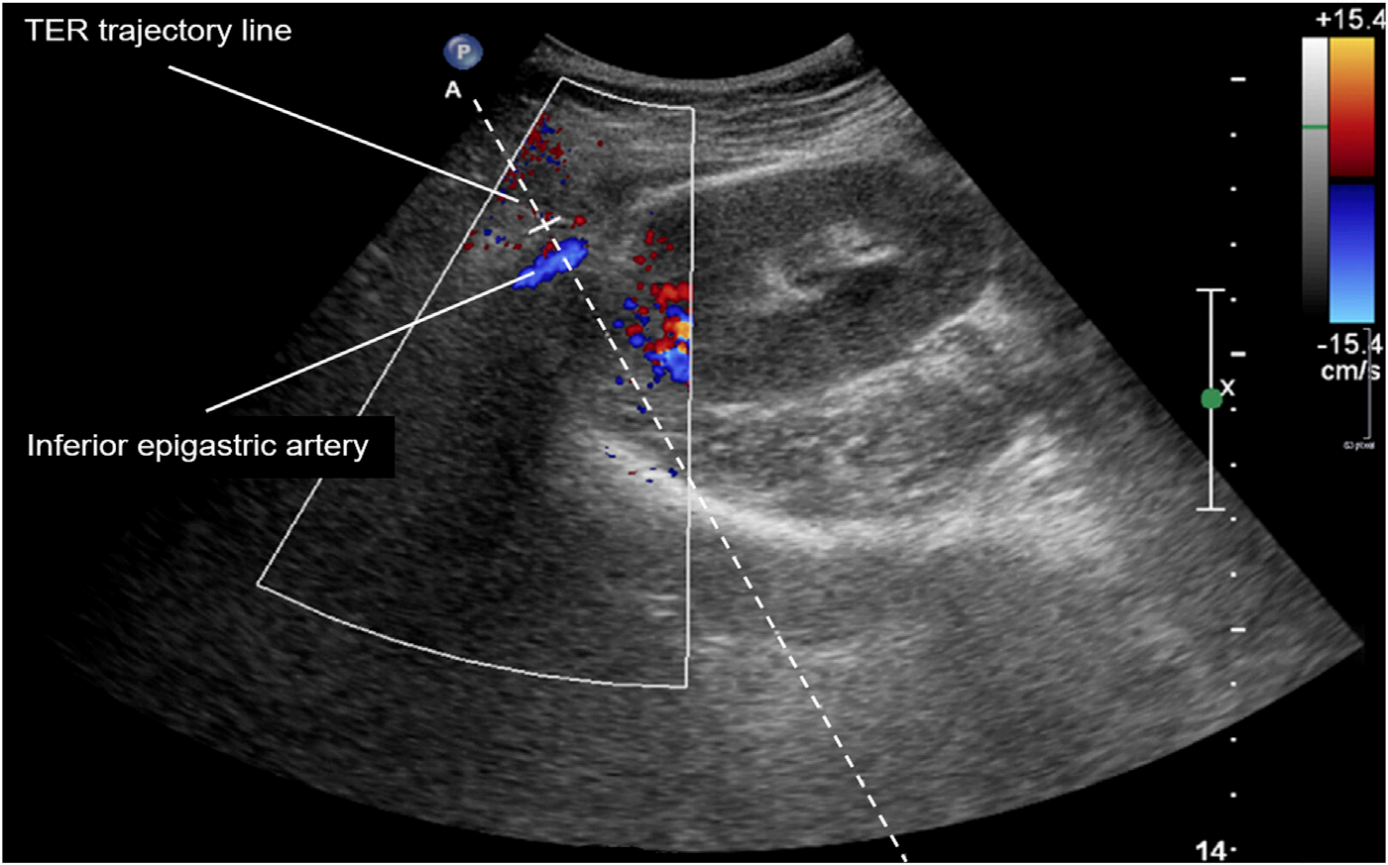
Color-duplex ultrasound image of right iliac kidney allograft and dislocated inferior epigastric artery. TER, tangential, extraperitoneal, retrorenal.

Sample adequacy rates range from 52.9 [20] to 99.5% [8] in the literature (Supplementary Table S4). However, some of the previous studies [5, 20, 27] regarded glomerular yield only, and thus, might overestimate adequacy. With an overall sample adequacy rate of 86.7% our study is among the top 6 studies reporting adequacy according to Banff classification. Adequacy rate increased to 94.1% in our study, if two or more core samples were analyzed. However, the latter applied to only 46.1% of biopsies due to frequent electron microscopic work-up of a second core sample. It is likely to speculate that adequacy rates would have exceeded 90% once these samples were analyzed. Based on this finding we now obtain three core samples in case of planned electron microscopic work-up. Biopsy technique and needle size vary among different studies, however, it has been previously stated that adequacy rates rather correlate with biopsy technique than needle size [24, 28] (Supplementary Table S4). As we found no significant difference between resident status and complication as well as adequacy rates (Supplementary Tables S1, S2), the novel TER biopsy approach can be considered appropriate for training biopsies. This is in contrast to a previous study stating a possible association of major complications with lesser operator experience in tangential allograft biopsy [2]. Previous studies evaluating tangential biopsy approaches [2, 3] did not stipulate a particular biopsy region or needle paths. However, transperitoneal needle paths are more likely to cause intraperitoneal hematoma and medial-to-lateral biopsy approaches are prone to injure both the rectus sheath and inferior epigastric artery with subsequent development of rectus sheath hematoma. Both complications have been previously reported with tangential biopsy approaches [2, 5]. With the TER approach these complications are less likely to occur as an exclusive extraperitoneal as well as lateral-to-medial biopsy approach keeps safe distance to both the rectus sheath, inferior epigastric artery, and the peritoneal fold. However, as the inferior epigastric artery might not be located in its usual position along the rectus abdominis muscle but dislocated further lateral due to mobilization during transplantation, the TER biopsy approach should be modified to avoid vascular injury in that case (Figure 3). While tangential cortex biopsy has been previously demonstrated to convey substantial advantages in terms of safety and adequacy [2, 3, 5], our results do not support the hypothesis that retrorenal biopsy approaches may lead to uncontrollable bleeding [5]. In contrast, the surrounding iliopsoas muscle as well as the dorsal pelvis rather serve as natural barriers against extended hematoma formation. Furthermore, the iliopsoas muscle is the only adjacent organ structure to be accidentally injured in case of a retrorenal biopsy approach. While previous studies inconsistently reported the number of biopsy attempts per biopsy and calculated complication rates per biopsy only, our study is the first to present complication rates per biopsy attempt and biopsy event. The present study is limited by a small sample size.

By demonstrating low major complication (<1%) and high sample adequacy rates (>90% when two or more samples are analyzed) our present study confirms high safety and efficacy of the novel TER approach for standard kidney transplant biopsy. Furthermore, this is the first study to 1) comprehensively report both major and minor complication rates based on a standardized post-procedural management and 2) confirm the eligibility of the TER approach for supervised training biopsies with respect to safety and efficacy.

## Capsule Summary Sentence

Ultrasound-guided percutaneous renal transplant biopsy is the gold-standard procedure for allograft pathology work-up. Recent studies, including previous research at our institution, postulate better safety and efficacy of tangential compared to radial approaches, however, there is no general consensus regarding biopsy needle path for this standard technique. In this context, we recently described a unified tangential, extraperitoneal, retrorenal (TER) approach for standard allograft biopsy and demonstrated excellent safety and efficacy in a pilot study (Transpl Int. 2017; 30: 947–50). By penetrating the allograft parallel to the renal capsule (tangential component), keeping safe distance to the peritoneal fold (extraperitoneal component) and targeting the posterior side of the allograft in a lateral-to-medial approach (retrorenal component), the TER approach aims at reducing the risk of intraperitoneal as well as rectus sheet hematoma. By verifying low major complication (<1%) and high adequacy (>90%) rates among 250 patients undergoing 330 kidney transplant biopsies our present study confirms safety and efficacy of the TER approach for standard ultrasound-guided allograft biopsy. Furthermore, this is the first study to (1) assess both major and minor complications based on a standardized post-procedural ultrasound follow-up as well as to (2) confirm the eligibility of TER kidney transplant biopsy for nephrology training.

## Data Availability

The raw data supporting the conclusions of this article will be made available by the authors, without undue reservation.

## Abbreviations

AVF, arteriovenous fistulas; CT, computer tomography; G, gauge; TER, tangential extraperitoneal retrorenal.
