# Supplementary material for: The Tangential Extraperitoneal Retrorenal Approach in Kidney Transplant Biopsy: An Observational Study to Assess Complication and Adequacy Rates
Source: Transpl Int. 2022 Jan 13;35:10068. doi: 10.3389/ti.2021.10068 (PMC8842347; doi:10.3389/ti.2021.10068)
Supplement: Supplementary file 1 [file DataSheet1.docx]

**Supplementary Material**

**Table S1:** Complications of kidney transplant biopsy based upon nephrology training status

|  | resident | | consultant | |
| --- | --- | --- | --- | --- |
|  | 249 biopsy attempts  (35.1) | 116 biopsies  (35.2) | 460 biopsy attempts  (64.9) | 214 biopsies  (64.9) |
| *Minor complications* | | | | |
| Superficial bleeding | 5^a^ (2.0) | 5^a^ (4.3) | 16^a^ (3.5) | 16^a^ (7.5) |
| Gross hematuria | 4 (1.6) | 4 (3.5) | 6 (1.3) | 6 (2.8) |
| AV-fistula | 3 (1.2) | 3 (2.6) | 15 (3.3) | 15 (7.0) |
| Subcapsular hematoma | 3^a^ (1.2) | 3^a^ (2.6) | 4 (0.9) | 4 (1.9) |
| Perinephric hematoma | 34^b^ (13.7) | 34^b^ (29.3) | 48^a^ (10.4) | 48^a^ (22.4) |
| Deep vein thrombosis | 0 (0.0) | 0 (0.0) | 1 (0.2) | 1 (0.5) |
| Vasovagal reaction | 2 (0.8) | 2 (1.7) | 3 (0.7) | 3 (1.4) |
| Hypertensive urgency | 0 (0.0) | 0 (0.0) | 1 (0.2) | 1 (0.5) |
| Drainage of serous fluid | 0 (0.0) | 0 (0.0) | 2 (0.4) | 2 (0.9) |
| Pain | 0 (0.0) | 0 (0.0) | 2^a^ (0.4) | 2^a^ (0.9) |
| Total ^§^ | 51 (20.5) | 51 (44.0) | 98 (21.3) | 98 (45.8) |
| *Major complications* | | | | |
| Catheter for gross hematuria | 1 (0.4) | 1 (0.9) | 0 (0.0) | 0 (0.0) |
| Transfusion | 1^a^ (0.4) | 1^a^ (0.9) | 1 (0.2) | 1 (0.5) |
| Interventional radiology with embolization | 1^a^ (0.4) | 1^a^ (0.9) | 0 (0.0) | 0 (0.0) |
| Total ^§^ | 3 (1.2) | 3 (2.6) | 1 (0.2) | 1 (0.5) |

Data are displayed as number (percent).

^a^ One modified TER biopsy.

^b^ Three modified TER biopsies.

^§^ no significant association of resident training status with major and minor complications

**Table S2:** Sample adequacy of kidney transplant biopsy based upon nephrology training status

|  | resident | consultant |
| --- | --- | --- |
| No. of biopsies | 116 (35.2) | 214 (64.9) |
| No. of biopsies considered  Adequate  Minimal  Inadequate  ND  Adequate and minimal ^§^ | 69 (59.5)  33 (28.5)  14 (12.1)  0 (0.0)  102 (87.9) | 123 (57.5)  61 (28.5)  28 (13.1)  2 (0.9)  184 (86.0) |

Data are displayed as number (percent). ND = no data.

^§^ no significant association of resident training status with sample adequacy

**Table S3:** Kidney transplant biopsy studies reporting major complication rates

|  | No. of US guided kidney allograft biopsies / biopsy attempts | Biopsy technique | Gross hematuria | Arteriovenous fistula | Perinephric  and/or  subcapsular hematoma | Transfusion | Surgery   and/or  interventional radiology  with/without embolization | Loss of graft | Loss of life | major complication rate |
| --- | --- | --- | --- | --- | --- | --- | --- | --- | --- | --- |
| **Pirklbauer et al. ^a^** | 330 / 709 | RT-US | 3.0 / 1.4 (10) | 5.5 / 2.5 (18) | 27.0 / 12.6 (89) | 0.6 / 0.3 (2) | 0.3 / 0.1 (1) | 0.0 / 0.0 (0) | 0.0 / 0.0 (0) | 1.2 / 0.6 (4) |
| **Pirklbauer et al. ^a^**  (TER only) | 317 / 683 | RT-US | 3.2 / 1.4 (10) | 5.7 / 2.6 (18) | 26.5 / 12.3 (84) | 0.3 / 0.2 (1) | 0.0 / 0.0 (0) | 0.0 / 0.0 (0) | 0.0 / 0.0 (0) | 0.6 /0.3 (2) ^b^ |
| Baffour et al. ^1^ | 6700 | RT-US | 0.3 (21) | NR | 0.3 (23) | 0.2 (11) ^c^ | 0.2 (16) ^d^ | 0.0 (0) | 0.0 (0) | 0.2 (16) |
| Patel et al. ^2^ | 3838 | RT-US | NR | NR | NR | NR | NR | 0.0 (0) | 0.0 (0) | 0.3 (10) ^e^ |
| Redfield et al. ^3^ | 3738 | RT-US | 0.0 (0) | 0.1 (4) | 0.5 (18) | 0.7 (26) | 0.4 (15) | 0.0 (0) | 0.0 (0) | 0.4 (15) ^f^ |
| Morgan et al. ^4^ | 2514 | RT-US | 0.1 (2) ^g^ | 0.1 (2) ^g^ | 0.6 (15) ^g^ | 1.3 (33) | 0.7 (18) | 0.0 (0) | 0.0 (0) | 1.9 (47) |
| Furness et al. ^5^ | 2127 ^g^  1486 ^h^ | US or CT guidance | 2.8 (41) | 2.4 (36) | 2.6 (39) | 0.1 (3) | 0.1 (3) | 0.1 (1) | 0.0 (0) | 0.4 (8) |
| Schwarz et al. ^6^ | 1670 | US guidance | 3.5 (58) | 7.3 (122) | 2.5 (41) | 0.3 (5) | 0.0 (0) | 0.0 (0) | 0.0 (0) | 1.0 (17) |
| Schmid et al. ^7^ | 57 ^i^  1614 | transfemoral  US guidance | 17.5 (10)  3.2 (52) | 8.8 (5)  5.9 (95) | 1.8 (1)  1.2 (19) | 0.0  0.1 (2) | 1.8 (1)  0.2 (4) | 0.0 (0)  0.0 (0) | 0.0 (0)  0.0 (0) | 1.8 (1)  0.4 (6) |
| Li et al. ^8^ | 880 | RT-US | NR | NR | NR | NR | NR | NR | NR | 1.1 (10) |
| Tapia-C. et al. ^9^ | 390 | US-guidance | 0.8 (3) | 3.6 (14) | 3.3 (13) | 3.1 (12) | 1.8 (7) | 0.3 (1) | 0.5 (2) | 5.6 (22) |
| Torres-R. et al. ^10^ | 385 | RT-US | 1.0 (4) | 0.0 (0) | 2.3 (9) | 0.0 (0) | 0.5 (2) | 0.0 (0) | 0.0 (0) | 0.5 (2) |
| Patel et al. ^11^ | 294 | RT-US | 0.7 (2) | NR | 0.7 (2) ^j^ | 0.7 (2) | 0.0 (0) | 0.0 (0) | 0.0 (0) | 0.7 (2) |
| Ho et al. ^12^ | 195 | RT-US | 4.1 (8) | 0.0 (0) | 3.6 (7) | 2.6 (5) | 0.5 (1) | 0.0 (0) | 0.0 (0) | 3.6 (7) |
| Shin et al. ^13^ | 188 | RT-US | NR | 1.1 (2) | 1.6 (3) | 0.0 (0) | 0.0 (0) | 0.0 (0) | 0.0 (0) | 0.0 (0) |
| Yablon et al. ^14^ | 124 | RT-US | 4.8 (6) | 0.0 (0) | 1.6 (2) ^k^ | 0.8 (1) | 1.6 (2) | 0.0 (0) | 0.0 (0) | 3.2 (4) |

**Legend Table S3:**

| Study design | |
| --- | --- |
| Single-center, retrospective: ^1-4,7,9-13^  Single-center, prospective: ^8,14^  Single-center, retrospective and prospective: ^6^  Multi-center, retrospective: ^5^ | |
| Information | Abbreviation |
| data are displayed as percent (numbers)  ^a^ complication rates calculated for biopsy events / biopsy attempts  ^b^ including one rinsing catheter for gross hematuria  ^c^ perinephric bleeding requiring angiography with or without embolization and/or blood transfusion  ^d^ including hematuria-related continuous bladder irrigation with or without percutaneous nephrostomy  ^e^ defined as CTCAE grade 3  ^f^ defined as CTCAE grade 3 and 4  ^g^ biopsies evaluated for major complications only  ^h^ biopsies evaluated for minor complications only  ^i^ transfemoral biopsy approach  ^j^ including one intraperitoneal hematoma  ^k^ two intraperitoneal hematomas | NR = not reported  No. = number  RT-US = real-time ultrasound  US = ultrasound  TER = tangential extraperitoneal retrorenal |
| only studies reporting major complications were considered | |

**Table S4:** Kidney transplant biopsy studies reporting sample adequacy

|  | Needle  size (G) | Core  sample (no.) | Adequacy (%)  Banff criteria | Biopsy technique |
| --- | --- | --- | --- | --- |
| **Pirklbauer et al.** | 16 | 2 | 86.7  Adequate + minimal | RT-US  TER approach |
| Morgan et al. ^4^ | 18 | 2 | 99.5  Adequate + minimal | RT-US  Cortical tangential approach |
| Schwarz et al. ^6^ | 16 | 1-2 | 76.0  Adequate + minimal | US-guidance |
|  | 18 |  | 53.0  Adequate + minimal |  |
| Schmid et al. ^7^ | 19 | ≥ 3 | 50.9  Adequate | Transfemoral approach |
|  | 16, 18 | 2 | 54.9  Adequate | US-guidance |
| Fang et al. ^15^ | 16, 18 | 1-3 | 98.8  Adequate | US-guidance |
| Torres-R. et al. ^10^ | 16 | 2-3 | 85.5  Adequate + minimal | RT-US |
| Patel et al. ^11^ | 18 | 1-3 | 95.2 ^a^  Adequate + minimal | RT-US  Cortical tangential approach |
| Laute et al. ^16^ | 16 | 2 | 70.0  Adequate + minimal | RT-US |
| Plattner et al. ^17^ | 16, 18 | 2.5 ± 0.6  (mean ± SD) | 89.0  Adequate + minimal |  |
|  |  |  | 84.6  Adequate | US-L |
|  |  |  | 86.8  Adequate | RT-US |
|  |  |  | 69.6  Adequate | US-T |
| Shin et al. ^13^ | 18 | 2 | 95.2 ^b^  Adequate + minimal | RT-US  Cortex-only view |
| Birk et al. ^18^ | 16 | 2 | 96.0 ^c^  Adequate | US-L |
| Reschen et al. ^19^ | 18 | 2.4 ^d^  (mean) | 68.2 ^b^  Adequate | US-guidance |
| Nicholson et al. ^20^ | 14, 16, 18 | NR | 71.0 ^b^  Minimal | RT-US |
|  | 14 |  | 85.9 ^b^  Minimal |  |
|  | 16 |  | 75.8 ^b^  Minimal |  |
|  | 18 |  | 52.9 ^b^  Minimal |  |

**Legend Table S4:**

| Abbreviation | | Information |
| --- | --- | --- |
| G  NR  no.  RT-US  US  US-L  US-T | = gauge  = not reported  = number  = real-time ultrasound  = ultrasound  = ultrasound-localized  = ultrasound-trocar | ^a^ data available for 98.6 % of biopsies  ^b^ arteries not considered  ^c^ data available for 94.7 % of biopsies  ^d^ data available for 39.3 % of biopsies |
| only studies reporting sample adequacy according to Banff criteria were considered | | |

**Supporting information references**

1. Baffour FI, Hickson LJ, Stegall MD, et al. Effects of Aspirin Therapy on Ultrasound-Guided Renal Allograft Biopsy Bleeding Complications. *J Vasc Interv Radiol.* 2017;28(2): 188-194.

2. Patel MD, Young SW, Scott Kriegshauser J, Dahiya N. Ultrasound-guided renal transplant biopsy: practical and pragmatic considerations. *Abdom Radiol (NY).* 2018;43(10): 2597-2603.

3. Redfield RR, McCune KR, Rao A, et al. Nature, timing, and severity of complications from ultrasound-guided percutaneous renal transplant biopsy. *Transpl Int.* 2016;29(2): 167-172.

4. Morgan TA, Chandran S, Burger IM, Zhang CA, Goldstein RB. Complications of Ultrasound-Guided Renal Transplant Biopsies. *Am J Transplant.* 2016;16(4): 1298-1305.

5. Furness PN, Philpott CM, Chorbadjian MT, et al. Protocol biopsy of the stable renal transplant: a multicenter study of methods and complication rates. *Transplantation.* 2003;76(6): 969-973.

6. Schwarz A, Gwinner W, Hiss M, Radermacher J, Mengel M, Haller H. Safety and adequacy of renal transplant protocol biopsies. *Am J Transplant.* 2005;5(8): 1992-1996.

7. Schmid A, Jacobi J, Kuefner MA, et al. Transvenous Renal Transplant Biopsy via a Transfemoral Approach. *Am J Transplant.* 2013;13(5): 1262-1271.

8. Li CH, Traube LE, Lu DS, et al. Implementation and Results of a Percutaneous Renal Allograft Biopsy Protocol to Reduce Complication Rate. *J Am Coll Radiol.* 2016;13(5): 549-553.

9. Tapia-Canelas C, Zometa R, López-Oliva MO, et al. [Complications associated with renal graft biopsy in transplant patients]. *Nefrologia.* 2014;34(1): 115-119.

10. Torres-Rodríguez IB, Castella-Fierro E, Serres-Creixans X, et al. Safety and efficacy of outpatient biopsy in renal transplantation. *Nefrologia.* 2014;34(6): 749-755.

11. Patel MD, Phillips CJ, Young SW, et al. US-guided renal transplant biopsy: efficacy of a cortical tangential approach. *Radiology.* 2010;256(1): 290-296.

12. Ho QY, Lim CC, Thangaraju S, et al. Bleeding Complications and Adverse Events After Desmopressin Acetate for Percutaneous Renal Transplant Biopsy. *Ann Acad Med Singap.* 2020;49(2): 52-64.

13. Shin J, Park SY. Diagnostic efficacy and safety of ultrasound-guided kidney transplant biopsy using cortex-only view: a retrospective single-center study. *Eur Radiol.* 2019;29(10): 5272-5279.

14. Yablon Z, Recupero P, McKenna J, Vella J, Parker MG. Kidney allograft biopsy: timing to complications. *Clin Nephrol.* 2010;74(1): 39-45.

15. Fang J, Li G, Xu L, et al. Complications and clinical management of ultrasound-guided renal allograft biopsies. *Transl Androl Urol.* 2019;8(4): 292-296.

16. Laute M, Vanholder R, Voet D, et al. Safety and sample adequacy of renal transplant surveillance biopsies. *Acta Clin Belg.* 2013;68(3): 161-165.

17. Plattner BW, Chen P, Cross R, Leavitt MA, Killen PD, Heung M. Complications and adequacy of transplant kidney biopsies: A comparison of techniques. *J Vasc Access.* 2018;19(3): 291-296.

18. Birk PE, Blydt-Hansen TD, Dart AB, Kaita LM, Proulx C, Taylor G. Low incidence of adverse events in outpatient pediatric renal allograft biopsies. *Pediatr Transplant.* 2007;11(2): 196-200.

19. Reschen ME, Mazzella A, Sharples E. A retrospective analysis of the utility and safety of kidney transplant biopsies by nephrology trainees and consultants. *Ann Med Surg (Lond).* 2018;28: 6-10.

20. Nicholson ML, Wheatley TJ, Doughman TM, et al. A prospective randomized trial of three different sizes of core-cutting needle for renal transplant biopsy. *Kidney Int.* 2000;58(1): 390-395.
